# Natural Variation of Model Mutant Phenotypes in *Ciona intestinalis*

**DOI:** 10.1371/journal.pone.0002344

**Published:** 2008-06-04

**Authors:** Paolo Sordino, Nikos Andreakis, Euan R. Brown, Nicola I. Leccia, Paola Squarzoni, Raffaella Tarallo, Christian Alfano, Luigi Caputi, Palmira D'Ambrosio, Paola Daniele, Enrico D'Aniello, Salvatore D'Aniello, Sylvie Maiella, Valentina Miraglia, Monia Teresa Russo, Gerarda Sorrenti, Margherita Branno, Lucio Cariello, Paola Cirino, Annamaria Locascio, Antonietta Spagnuolo, Laura Zanetti, Filomena Ristoratore

**Affiliations:** 1 Laboratory of Biochemistry and Molecular Biology, Stazione Zoologica Anton Dohrn, Naples, Italy; 2 Laboratory of Neurobiology, Stazione Zoologica Anton Dohrn, Naples, Italy; 3 Physiopathology and Human Reproduction, “G. Rummo” Hospital, Benevento, Italy; 4 Tigem-Telethon Institute of Genetics and Medicine, Naples, Italy; 5 Department of Genetics, University of Barcelona, Barcelona, Spain; 6 Immunorégulation, Institut Pasteur, Paris, France; 7 Institute of Marine Science, C.N.R., Lesina, Italy; 8 Service of Marine Resources for Research, Stazione Zoologica Anton Dohrn, Naples, Italy; Katholieke Universiteit Leuven, Belgium

## Abstract

**Background:**

The study of ascidians (Chordata, Tunicata) has made a considerable contribution to our understanding of the origin and evolution of basal chordates. To provide further information to support forward genetics in *Ciona intestinalis*, we used a combination of natural variation and neutral population genetics as an approach for the systematic identification of new mutations. In addition to the significance of developmental variation for phenotype-driven studies, this approach can encompass important implications in evolutionary and population biology.

**Methodology/Principal Findings:**

Here, we report a preliminary survey for naturally occurring mutations in three geographically interconnected populations of *C. intestinalis*. The influence of historical, geographical and environmental factors on the distribution of abnormal phenotypes was assessed by means of 12 microsatellites. We identified 37 possible mutant loci with stereotyped defects in embryonic development that segregate in a way typical of recessive alleles. Local populations were found to differ in genetic organization and frequency distribution of phenotypic classes.

**Conclusions/Significance:**

Natural genetic polymorphism of *C. intestinalis* constitutes a valuable source of phenotypes for studying embryonic development in ascidians. Correlating genetic structure and the occurrence of abnormal phenotypes is a crucial focus for understanding the selective forces that shape natural finite populations, and may provide insights of great importance into the evolutionary mechanisms that generate animal diversity.

## Introduction

The primary objective of forward genetics is to provide a low-cost resource for mutants with interesting phenotypes that may be subsequently mapped and identified at the molecular level [1]. Historically, records of mutant phenotypes have been the driving force that has linked biological functions to specific molecular mechanisms. In developmental biology, phenotypic traits of interest are explored by documenting the physical consequences of gene alteration, and then uncovering the molecular nature by positional cloning or gene candidate approaches. This concept applies however to a very limited number of model species in which mutagenesis techniques allow us to compare phenotype and genotype in great detail. It is therefore relevant to investigate the potential of naturally occurring mutations in traditional and non-traditional model organisms which are easy to collect and rear, and are amenable to genetic studies by breeding schemes [2].

Mutant alleles occur in natural populations and are able to influence individual fitness differently. Genetic drift acts upon these alleles by causing rapid changes in their frequencies according to the rate with which these mutations are filtered by natural selection. This indicates that a high percentage of individuals should be free of lethal mutations and carry early-stage positively selected phenotypes. Mutational pressure coupled with random sampling drift contributes significantly to the genetic load, inbreeding depression and finally to the pool of genetic variability of a population [3], [4]. Frequency distribution of naturally occurring mutations is often associated with geographical and environmental factors able to affect genetic patterning and demography. In evolutionary genetics of populations, new mutations may insignificantly affect the phenotype and appear to be nearly neutral with respect to natural selection or, they may be clearly deleterious and immediately eliminated from the population. The occurrence of deleterious alleles with observable phenotypic effects is not only an essential component for the interpretation of mutation-selection equilibrium rates in biological finite populations, but also for gene and gene function discovery. Our hypothesis is that most of the accumulation and distribution of spontaneous mutant classes will depend upon population parameters, including effective population size, genetic variability, reproductive strategies and geographical barriers.

Numerous advances, including genome sequencing, have considerably enhanced the use of the model tunicate, *Ciona intestinalis* (L., 1767) (Ascidiacea, Cionidae) in studies of development and evolution in Chordata [5]–[8]. *C. intestinalis* is a filter-feeding hermaphrodite invertebrate species that lives in shallow eutrophic waters, such as estuaries, lagoons and marinas, with a geographical distribution that spans most temperate to sub-boreal zones of both hemispheres. *C. intestinalis* forms dense coastal aggregates of ecological and economical relevance [9], whose distribution in space and time is explained by the interaction of factors as predation and recruitment (ascidian larvae have low dispersal capability) [10]. It appears that the Linnean taxon consists of at least two genetically and sexually separated, but morphologically similar species, that have been preliminarily named sp. A, inhabiting temperate waters worldwide, and sp. B, restricted to the north Atlantic and North Sea [11]–[13]. *Ciona* genomes analyzed so far display a high degree of DNA polymorphism, possibly associated with a large effective population size [14]–[18]. It has been shown that the genetic diversity of *C. intestinalis* sp. A ranges mostly within sites among regions, whereas population structuring appears to be higher in sp. B than in sp. A [12], [19]–[22].

In the wild, the species is characterized by seasonal fluctuations in demography. *Ciona*, as other ascidians, accumulates toxic substances by acting as a biological filter for the purification of coastal waters and consequently as an important bio-indicator [23], [24]. In shallow hard substrata of highly transformed environments, major ecological variables such as the concentration of pollutants and high temperature affect negatively the species demography. Densely structured colonies exhibit a seasonal decline in their population size characterized by cyclic population reduction to extinction and preferential recolonization [25]. The origins and genetic structure of these newly established populations are not entirely clear. Yet, this seasonal dynamics represents an open question with respect to the recolonization mechanisms and the importance of local environmental factors such as hydrodynamism and food sources able to promote recruitment of new individuals in a given site.

Random germline mutagenesis has been pursued by means of chemical [26]–[30] and transgenic [31]–[35] technologies of limited mutational activity. Currently, *Minos*-specific electroporation and remobilization techniques are expected to provide higher proficiency and amenability of insertional mutagenesis screenings [36], [37]. Generation of novel models for studying specific biological aspects could exploit naturally occurring variation [30], [38], [39]. To explore the details of how to profit from spontaneous mutations in ascidians, we hereby present a screen for visible developmental phenotypes in *C. intestinalis* sp. A populations from interconnected geographical locations. Twelve microsatellite loci were used to investigate the genetic structure of these populations and to compare it with rates and categories of the mutations encountered in sampling sites. Early emphasis on two mutant lines (*omero* and *curly*) provides model systems for addressing determination of sensory organs and neuro-muscular activity.

## Results

### Screening

We used a simple F_1_ scheme based on artificial self-fertilization in order to identify deleterious alleles acting during *C. intestinalis* sp. A embryogenesis. As already shown in zebrafish, a morphology-based mutation screen can isolate potentially important phenotypes [40]. Interest in early developmental mechanisms restricted our focus to pre-metamorphic stages (∼1 day screening time), complex developmental traits like metamorphosis and organogenesis were not surveyed. Individuals were sampled from three locations (VC, Villaggio Coppola; FuI, Lake Fusaro; CdS, Castellamare di Stabia) around the Bay of Naples (Thyrrenian Sea, Italy), in an area of intense human activity [12]. Population size and density is quite stable through the years, except for the period from July to September, when rise in temperature and primary productivity leads to oxygen depletion (see Introduction). Otherwise, sexually mature specimens were collected every week from January 2004 to May 2006. The oversampling was prevented by collecting specimens from one population *per* month.

Of a total of 370 adult animals analyzed in this study, 118, 151 and 101 originated from VC, FuI and CdS, respectively. The rates of spawning (78.8–89.0% among populations), self-fertility (74% of all individuals; 17.9–33.2% within broods) and reproductive capacity (∼1700–3000 eggs *per* individual) gave rise to a high percentage (54.3–69.5%) of adults suitable for phenotype screening in F_1_ progeny (>30 early blastulae) (Table 1). In addition, the task was rendered easier by the higher rates of self-fertilization during the winter [41]. After the first self-fertilization test, candidate heterozygote carriers were rested for two weeks while animal health was observed. After this period, individuals carrying gametes in both gonoducts were used for validation tests by inheritance or repeated selfing.

**Table 1:** Screening of Ciona intestinalis sp. A populations for naturally occurring mutations.

	VC	FuI	CdS
**Sample size**	118	151	101
**Spawning events**	89.0	78.8	85.1
**Selfing events**	69.5	54.3	62.4
**Mutations**	19.5	13.4	15.8
**Lethals**	50.0	18.2	60.0
**Non-lethals**	50.0	81.8	40.0

Numbers of individuals analyzed in this study, and the percentages of (i) spawning events, (ii) self-fertilized clutches with at least 30 blastulae, (iii) mutation carriers over individuals suitable for screening and (iv) fractions of lethal and non-lethal phenotypes.

Visual and behavioural analysis led to the identification of 37 mutant phenotypes affecting embryo morphology and motility in a stereotyped way typical of recessive alleles (Table 2). Variation of phenotype strength may explain frequencies of mutant alterations sharply below 25% within some crosses (*curly*, *levi*, *smerz*) (Table 2). Whereas these were much higher than any background rate of defects due to gamete quality or other epigenetic factors, the genetic causality was supported by transmission to the outcross progeny in all examined cases (9), and by repeated self-fertilization (28). Among individuals screened in this study, 16.3% were likely carriers of heterozygotic mutations. This value was highly variable among populations, with 19.5%, 15.8% and 13.4% of stereotyped abnormal phenotypes in VC, CdS and FuI sampling sites, respectively (Table 1).

**Table 2:** List of naturally occurring mutations identified during the screening.

Mutation	Pop	%	Phenotypic class	Description	Validation
*albus*	VC	27	sensory organs	No pigment	inheritance
*albus3*	VC	25	sensory organs	Weakly or no pigmented Ot	inheritance
*kashmir*	VC	25	sensory organs	No pigment	rescreen
*omero*	VC	25	sensory organs	No Ot, no Oc pigment	inheritance
*pale*	VC	23	sensory organs	Weakly pigmented Oc	inheritance
*shiva*	VC	28	sensory organs	Larger, often split, Oc pigmentation	inheritance
*bermuda*	VC	25	trunk	Small, abnormal endoderm	rescreen
*chiodino*	VC	28	trunk	Small round	rescreen
*verlaine*	VC	23	trunk	Anterior truncation	rescreen
*halftail*	VC	25	tail	Short, non-vacuolated Nc cells	rescreen
*streveza*	VC	25	tail	Short, non-intercalated Nc cells	rescreen
*curly*	VC	20	motility	Circling movement	rescreen
*fuoriditesta*	VC	25	multiple	External SV, short tail	inheritance
*pigtail*	VC	27	multiple	Small trunk, short tail	rescreen
*smerz*	VC	20	multiple	External SV, bent tail	rescreen
*camus*	VC	25	cell death	Whole body	rescreen
*albus2*	FuI	25	sensory organs	No pigment	rescreen
*bukowski*	FuI	23	sensory organs	No pigment	inheritance
*miller*	FuI	25	sensory organs	Supranumerary Ot	rescreen
*monkey*	FuI	25	sensory organs	Two Ot, no Oc pigment	rescreen
*pascoli*	FuI	26	sensory organs	No ocellus pigment	rescreen
*tasso*	FuI	27	sensory organs	Weakly pigmented Ot, no Oc pigment	inheritance
*valery*	FuI	25	sensory organs	Weakly pigmented Ot, no Oc pigment	rescreen
*divine*	FuI	25	CNS	No brain cavity	inheritance
*mallarmé*	FuI	25	trunk	Small	rescreen
*pasolini*	FuI	25	tail	Bent	rescreen
*uritsa*	FuI	25	tail	Short	inheritance
*beef*	CdS	22	sensory organs	No Oc pigment	rescreen
*levi*	CdS	20	CNS	External SV	rescreen
*mezacapa*	CdS	25	trunk	Anterior truncation	rescreen
*calvino*	CdS	26	tail	Short	rescreen
*jumbo*	CdS	25	tail	Short	rescreen
*rohmer*	CdS	24	tail	Short bent	rescreen
*quasimodo*	CdS	25	multiple	External SV, no pigment, short tail	rescreen
*montale*	CdS	22	multiple	Small trunk, bent tail	rescreen
*pavese*	CdS	23	multiple	Small trunk, one pigmented SO, bent tail	rescreen
*shellac*	CdS	27	multiple	External S, small trunk, bent tail	rescreen

Mutation name, population of origin, percentage of F_1_ homozygosity, mutation class, short phenotype description and method of validation. Abbreviations: CNS, central nervous system; Nc, notochord; Oc, ocellus; Ot, otolith; SO, sensory organs; SV, sensory vesicle. See Materials and Methods for validation procedures.

### Phenotypes

Ascidian development includes a wide set of variable traits, defined merely as the morphological and behavioural features that are determined throughout the life cycle. When different natural genotypes of the same species are directly compared, phenotypic variation may be observed for particular cell types. Deleterious alleles were classified into seven phenotypic classes. The most frequent type of mutations showed abnormal development of the larval sensory organs (14, 37.8% of the total). Other categories were those affecting tail (7), multiple traits (7), trunk (5), brain patterning (2), cell death (1) and larval motility (1) (Table 2).

**Figure 1:**
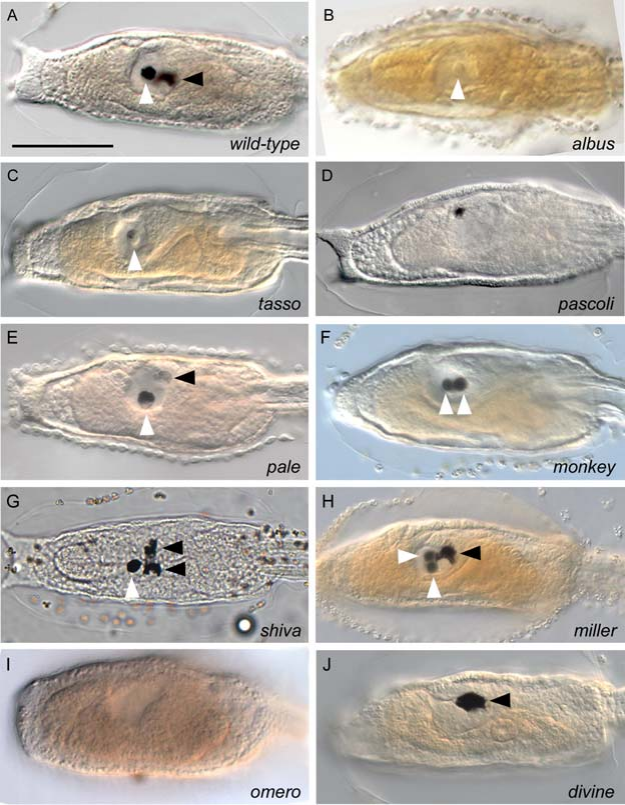
Homozygous mutant phenotypes with defects in the formation of sensory organs and brain vesicle. (A,B) Compared to wild-type larvae, no development of melanin is seen in the sensory organs of *albus* larvae. Scale bar, 100 µm. (C) Few pigment spots are observed in the otolith of *tasso*. (D) Only one undefined pigmented sensory organ is present in *pascoli*. (E) In *pale*, the ocellus is weakly pigmented, while the otolith is normal. (F) Supranumerary pigment cells in the otolith and none in the ocellus are observed in *monkey*. (G, H) Enlarged ocellus pigmentation and two otolith pigment cells occur in the *shiva* and *miller* mutants, respectively. (I) No pigmentation and at least no otolith are observed in *omero*. (J) The sensory vesicle of *divine* is not cavitated. White and black arrowheads indicate otolith and ocellus, respectively.

The chordate affiliation of *C. intestinalis* is exemplified by three main anatomical features: a dorsal nervous system, a notochord and gill slits [7], [42]. Located in dorsal-mid trunk position, the larval brain consists of a vesicle lined by ependymal and neuronal cell types, whose identity, function and fate are still debated [43], [44]. Two sensory organs containing pigment cells are visible in the brain (Figure 1A): the most anterior one, the otolith, is believed to function in geotactic responses and it is formed by a single pigment cell connected to the vesicle floor by a narrow stalk. The posterior organ, the ocellus, is involved in photoreception process. It is cup-shaped and composed of three lens cells, about 30 photoreceptors and one pigment cell. In all chordates, neural development is characterized by high conservation of cellular and molecular mechanisms [43], [45]. Stereotyped defects in distinct maturation steps of sensory organ development include albino phenotype (*e.g. albus*) (Figure 1B), few pigment spots limited to the otolith (*e.g. tasso*) (Figure 1C), accumulation of pigment restricted to a single undefined sensory organ (*e.g. pascoli*) (Figure 1D), normal otolith and reduced pigmentation on the ocellus (*pale*) (Figure 1E), two otolith and no ocellus pigment cells (*monkey*) (Figure 1F), larger or split ocellus pigmentation (*shiva*) (Figure 1G), supranumerary otolith pigment cells (*miller*) (Figure 1H) and absence of both otolith and ocellus pigment cells (*omero*) (Figure 1I). Other specific defects in the formation of the central nervous system are represented by the absence of a brain cavity in *divine* (Figure 1J), and by the externalised sensory vesicle occurring in *levi* and in three multiple phenotype mutants (Table 2).

**Figure 2:**
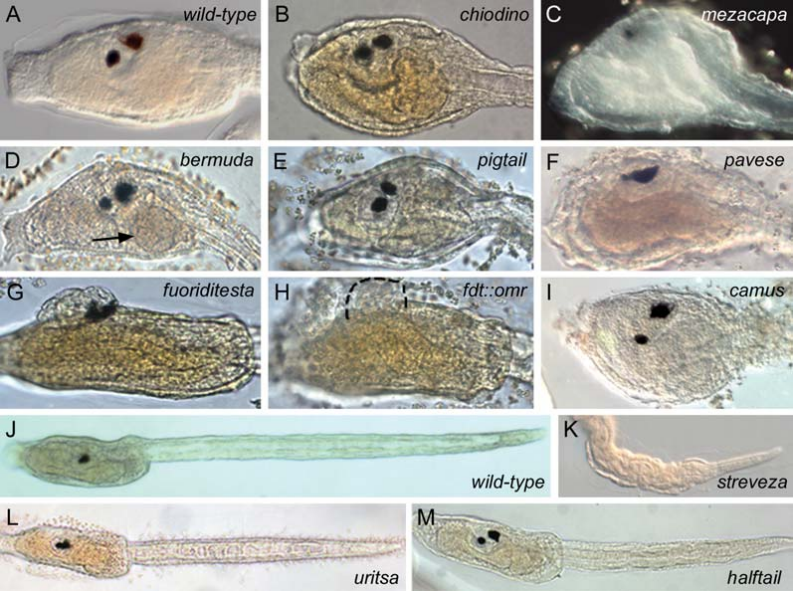
Examples of trunk, tail and multiple phenotypes. (A,B) Compared to the wild-type, the trunk and papillae of *chiodino* are smaller. (C) Anterior trunk and papillae do not form in *mezacapa*. (D) An endodermal structure is observed in *bermuda* that resembles a premature organ rudiment (black arrow). (E,F) The trunk is smaller in the multiple phenotype *pigtail* and *pavese* mutations. (G) *fuoriditesta* (*fdt*) is another multiple phenotype featuring a shorter tail (not shown) and an externalised sensory vesicle (white arrow). (H) *fdt* was originally isolated in a double heterozygosity carrier of *fdt* and *omero* mutations (see Figure 1) (dashed line delimits the sensory vesicle in double homozygous mutant larvae). (I) Massive cell death is observed across the body of *camus* larvae, as shown by the trunk. (J–M) Compared with wild-type larvae, some mutations display tails that are shorter than normal (L). The morphology of the notochord cells in the tails of *halftail* and *streveza* homozygous larvae suggests an impaired differentiation (K,M).

Besides containing the central nervous system, the larval trunk consists of an elaborated architecture composed of endoderm, mesenchyme cells, the peripheral nervous system and the sensory papillae [7] (Figure 2A). We identified several mutations with trunk defects at the larval stage: smaller-than-normal trunk (*e.g. chiodino*) (Figure 2B), anterior truncation (*e.g. mezacapa*) (Figure 2C), and the presence of a round-shaped structure resembling a premature digestive organ in the posterior endoderm (*bermuda*) (Figure 2D).

Phenotypes with multiple traits were also frequent, with abnormalities in trunk and tail (*e.g. pigtail*) (Figure 2E); trunk, tail and brain (*e.g. pavese*) (Figure 2F); and tail and brain (*e.g. fuoriditesta*) (Figure 2G). One double heterozygote carrier (*omero* and *fuoriditesta*) was isolated, as shown by independent allele segregation in F_2_ outcross generation (Figure 1I, 2G,H).

We identified seven broods with recessive alterations in tail formation (Figure 2J–M). Most of these phenotypes (6) share a shorter tail with a) normal anatomy (*e.g. uritsa*) (Figure 2L), b) kinked axis (*bell* and *rohmer*) (data not shown) and c) defects in notochord morphogenesis (*halftail* and *streveza*) (Figure 2K,M). Two defective phenotypes display tails of normal length with a kinked axis (*pasolini*) and abnormal tip (*junk*) (data not shown).

After hatching, normal larvae straighten out their tails and are then capable of symmetrical swimming by generating tail flicks (for orientation) and bilateral swimming movements of the tail [46]. It has always been presumed that the curved posture is as a result of the folding of the larva in the egg and that as the hatched larva matures, the notochord adopts a straight form. The *curly* phenotype was found to cause circling movements, likely due to defects in muscle contractions (see below).

Motility, trunk, brain, multiple and cell death categories (16 phenotypes, 43.2%) showed full lethality before settlement and metamorphosis. On the other hand, tail and sensory organ phenotypes were nearly viable (21, 56.8%), with inbred or outbred mutant homozygotes propagating until adulthood. Notably, the abundance of lethal- and non-lethal phenotypes varied sharply among populations, contributing a major portion in CdS (60%) and FuI (81.8%), respectively (Table 1,2).

### Characterization of a sensory organ mutant: omero

**Figure 3:**
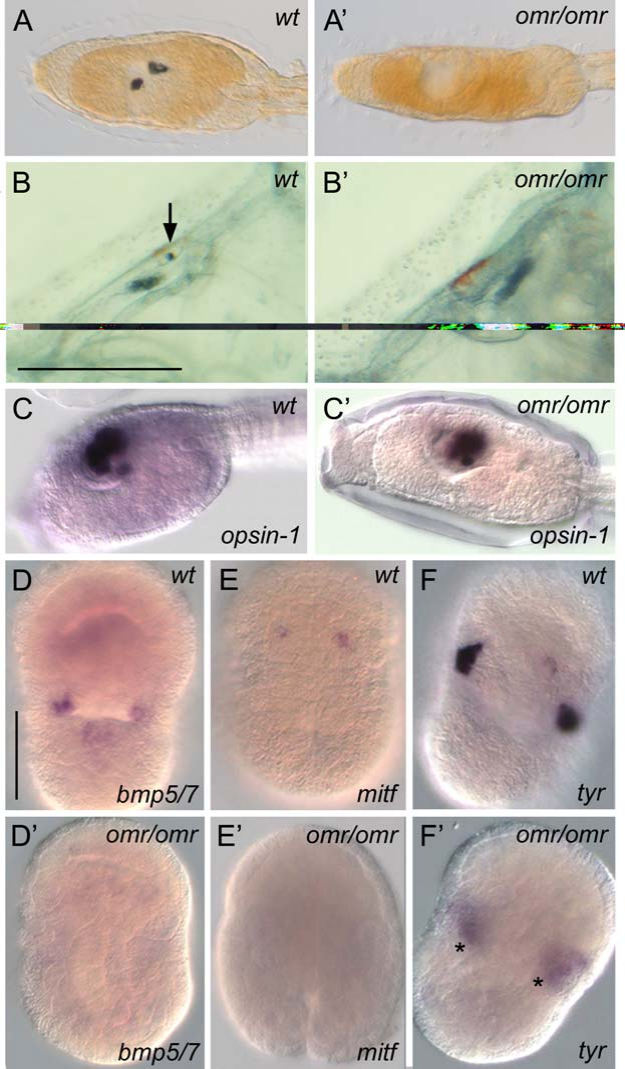
Morphological and molecular traits in wild-type and omero embryos. (A,B) Wild-type larvae and 2^nd^ juvenile ascidian stage (29 days post fertilization), respectively [41] (black arrow indicates pigment cells in the cerebral ganglion). Scale bar, 100 µm. (A′,B′) Corresponding stages in *omero* (white arrow points to lack of pigment in the brain). (C,C′) Expression of the photoreceptor marker *opsin-1* is unaffected at the larval stage. (D–F′) Conversely, transcriptional patterns of pigment cell specification genes are altered at neurula stage (asterisks highlight background staining in mesenchyme). Scale bar, 50 µm.

The *omero* (*omr*) mutant was initially identified due to the absence of the pigmented sensory structures (Figure 3A,A′). This mutant was capable of undergoing metamorphosis and to develop into a functionally normal juvenile (Figure 3B,B′). The founder was crossed to wild-type individuals, and the mutant line has been maintained for over five generations without any changes in phenotype. Further morphological characterization of *omr* mutant larva was effectuated by histological analyses of the larvae, allowing the confirmation of the total absence of the two pigment cells inside the sensory vesicle (data not shown). Mutant larvae were analysed by *in situ* hybridisation, in order to verify if the *omr* mutation was accompanied by abnormalities in sensory vesicle structure and by impairment of ocellus photoreceptors. The genes taken into account were the sensory vesicle markers *otx* and *six3/6*, and the photoreceptor markers *arrestin, opsin-1* and *pitx-2.* In comparison to control larvae, no detectable discrepancy in the expression of all the markers listed above was observed (see for example Figure 3C,C′). These results allow us to hypothesize that the mutation acts only on the formation of pigment cells and that neither the melanogenesis pathway nor other sensory vesicle differentiating processes are affected.

To investigate if the *omr* phenotype was caused by perturbation of early specification events, the expression patterns of genes known to be involved in neural tissue and pigment cell specification (*bmp5/7, mitf* and *tyr*) were examined in at neurula stage embryos. Although at this stage *omr* mutant embryos are morphologically indistinguishable from wild-type embryos, any alterations in gene expression should be evident in ∼25% of the embryos. The expression of *bmp5/7, mitf* and *tyr* is lost in the pigment cell precursors of 30%, 35% and 35% of embryos, respectively (Figure 3D–F′). These results suggest that the *omero* phenotype is due to changes in early pigment determinating cells. Hence, closer study of this mutant may provide new insights into the mechanisms that induce pigment cell formation.

### Characterization of a motility phenotype: curly

**Figure 4:**
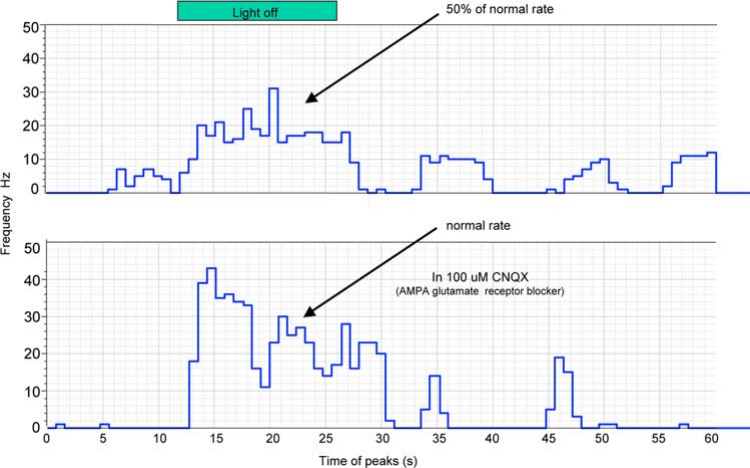
Physiological characterization of the curly mutation. Two graphs showing the effect of CNQX on the frequency of larval swimming. The histograms represent the mean frequency in 10 s bins of spikes before, during and after a light off stimulus.

The *curly* phenotype showed striking morphological and functional features. At larval stage, it maintained a curled posture for at least 48 hours after hatching (animals were observed over two days) and during this period made swimming movements and tail twitches which resulted in larvae describing roughly circular tracks. The curled ‘posture’ of the *curly* larvae is similar to that of freshly hatched normal larvae. No obvious malformation of the notochord was observed in the mutants at the light microscope level. Superficially they resembled larvae that have experimentally reversed L-R asymmetry due to one-sided expression of the nodal gene [47]. However, examining the phenotype in detail by recording from the muscles in the tail to see if there were any gross abnormalities in neuromuscular function, showed that these similarities were only superficial. Addition of curare (100 µM), a compound known to block cholinergic neuro-muscular transmission in tunicate larvae (*Halocynthia roretzi* [48]; *C. intestinalis*, E.R. Brown, unpublished data), blocked swimming but did not result in tail ‘straightening’ even after exposure for several hours. Suction electrode recordings from larval tails showed that muscle potentials were detected at half the normal frequency for both light off and spontaneous swimming bursts (20 vs 40; 10 vs 20 Hz respectively; Figure 4).

Addition of CNQX, an AMPA–like glutamate receptor blocker, instantaneously altered the swimming pattern producing normal muscle contraction frequencies and patterns. We speculate that the results are consistent with the idea that the *curly* mutant phenotype is due to the tonic action of a neuro-transmitter other than acetylcholine (possibly glutamate), which has a role in maintaining the shape adopted by the normal larvae before hatching, rather than gross developmental differences such as changes in LR symmetry [47]. In normal larvae the influence of this mechanism must be removed in the first 30 minutes or so after hatching.

### Population Genetics

**Figure 5:**
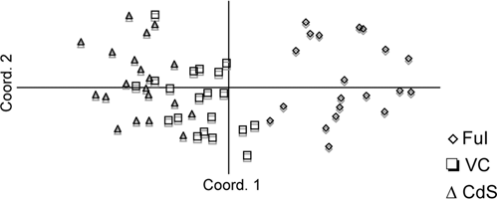
Principal coordinate analysis. Principal coordinate analysis shows significant differentiation between isolated (FuI) and open-sea (CdS/VC) populations.

It is assumed that the genetic variability of a population that is estimated from selectively neutral alleles is produced in a stepwise mode. This process is mathematically tested in locus-specific allelic frequencies in equilibrium conditions from natural finite random-mating populations [49], [50]. The statistics describing partition and distribution of genetic variability *per* locus and *per* population is summarized in Table 3. All microsatellite loci were found to be polymorphic with a total number of alleles *per* locus ranging from 3 to 24 (see Table 3). Hierarchical analysis of molecular variance revealed a moderate partition of genetic diversity among sampling sites across the entire dataset. Distribution of genetic differentiation was thus found to be higher within populations (87%, *P*<0.001) rather than between them (13%, *P*<0.001). Genetic distance from pairwise comparisons [51] and principal coordinate analysis showed significant differentiation only between CdS/VC and the FuI population (Table 4, Figure 5).

**Table 3:** Genetic characterization based on twelve nuclear microsatellite loci.

Locus	Allele	VC	FuI	CdS
Ci-1	*n/tot*	4/11	8/11	3/11
	*H_O_*	0.300	0.100	0.211
	*H_E_*	0.344	0.728	0.514
	*F*	0.127	0.863	0.590
	*p*	n.s.	***	n.s
Ci-2	*n/tot*	16/24	15/24	12/24
	*H_O_*	0.300	0.450	0.278
	*H_E_*	0.893	0.873	0.861
	*F*	0.664	0.484	0.677
	*P*	***	***	***
Ci-4	*n/tot*	3/3	3/3	3/3
	*H_O_*	0.250	0.200	0.500
	*H_E_*	0.501	0.486	0.645
	*F*	0.501	0.589	0.225
	*P*	*	**	*
Ci-5	*n/tot*	14/15	8/15	4/15
	*H_O_*	0.833	0.875	0.105
	*H_E_*	0.864	0.820	0.195
	*F*	0.036	−0.067	0.461
	*P*	*	n.s.	n.s.
Ci-7	*n/tot*	4/7	6/7	3/7
	*H_O_*	0.316	0.200	0.053
	*H_E_*	0.489	0.746	0.148
	*F*	0.354	0.732	0.645
	*P*	*	***	n.s.
Ci-8	*n/tot*	15/24	13/24	17/24
	*H_O_*	0.850	0.550	0.600
	*H_E_*	0.904	0.906	0.909
	*F*	0.059	0.393	0.340
	*P*	n.s.	***	***
Ci-9	*n/tot*	3/6	4/6	2/6
	*H_O_*	0.154	0.000	0.333
	*H_E_*	0.145	0.578	0.444
	*F*	−0.061	1.000	0.250
	*P*	n.s.	***	***
Ci-10	*n/tot*	14/21	14/21	12/21
	*H_O_*	0.714	0.850	0.900
	*H_E_*	0.903	0.858	0.868
	*F*	0.209	0.009	−0.037
	*p*	**	n.s.	n.s.
Ci-11	*n/tot*	6/8	5/8	4/8
	*H_O_*	0.235	0.611	0.050
	*H_E_*	0.651	0.736	0.444
	*F*	0.638	0.170	0.887
	*P*	***	n.s.	***
Ci-22	*n/tot*	6/13	9/13	7/13
	*H_O_*	0.750	1.000	0.500
	*H_E_*	0.570	0.770	0.660
	*F*	−0.316	−0.299	0.242
	*P*	n.s.	*	n.s.
Ci-49	*n/tot*	2/3	3/3	2/3
	*H_O_*	0.300	0.300	0.333
	*H_E_*	0.375	0.266	0.444
	*F*	0.200	−0.127	0.250
	*P*	n.s.	n.s.	n.s.
Ci-67	*n/tot*	6/8	6/8	6/8
	*H_O_*	0.750	0.900	0.650
	*H_E_*	0.791	0.769	0.620
	*F*	0.052	−0.171	−0.048
	*P*	n.s.	***	n.s.

* ***p*** **<0.05;**

** ***p*** **<0.01;**

*** ***p*** **<0.001**

n/tot, number of alleles *per* locus and *per* population over the total number of alleles *per* locus; H_O_, observed heterozygosis; H_E_, expected heterozygosis; F, fixation index; *P*, probability level for Hardy-Weinberg equilibrium; n.s., non significant.

**Table 4:** Genetic distance and gene flow among sites.

	VC	FuI	CdS
**VC**	0.000	2.699	4.760
**FuI**	0.404	0.000	0.603
**CdS**	0.154	1.881	0.000

Pairwise population comparison based on Nei [47] genetic distances in lower triangle matrix; Nm (estimated number of migrants) among populations in upper triangle matrix.

The genetic separation among sites as well as gene flow levels were not concordant with population geographic locations and distances (Table 4): VC and CDS sampling sites showed elevated levels of gene flow (Table 4, Nm = 4.76) and they were found to share both identical alleles for more than one locus and identical individual genotypes. Yet, specimens sampled within the aforementioned sites were found to be significantly distinct from one another.

Fisher's pair-wise exact tests for linkage disequilibrium performed considering all loci combinations were not significant (*p*>0.05) in any of the populations, indicating independent assortment of alleles at all loci. Observed genotypic proportions deviated significantly from Hardy-Weinberg (HW) expectations at several loci mainly for the FuI population, suggesting that HW proportions may be altered by small population size, non-random mating and inbreeding.

Taken together, the results indicated that the FuI population stands out from the whole data set in terms of levels of genetic distance, private alleles frequency and gene flow level (Table 3), demonstrating that this site is significantly differentiated from the others.

## Discussion

Artificial mutagenesis is a powerful approach to study developmental, physiological, or pathological processes, as a forward genetic complement in defining novel molecular functions. However, this technique is available only for a small fraction of animal and vegetal species with strong genetics. Since *C. intestinalis* is an ideal model organism for positional cloning of developmental mutations, we pointed to the contribution of natural genetic variation in large-scale investigations of mutant phenotypes. Here, we describe 37 anatomical and behavioural disorders that are exposed in the F_1_ generation as recessive alleles. The magnitude of individuals carrying mutant phenotypes (∼0.13–0.19) is consistent with previous estimates (∼20%) [30], and is apparently similar to that observed in other invertebrates such as *Drosophila* [reviewed in 52]. Information on the frequency and selective impact as inferred from genome-specific rates of mutation to deleterious alleles has been extensively gathered in only a few model organisms [*e.g.* 52]–[54]. It is known that a dynamic equilibrium occurs between the varying influences of several combined forces such as mating system, gene flow, purifying selection, inbreeding depression and heterozygosity within a population [2], [55]. However, mutation percentages and composition have never been connected to biogeographical constraints and demographic processes related to neutral genetic variability. The loss of coordination among these parameters together with the influence of habitat contamination and degradation may generate differences in sets of mutational variants and influence their frequencies [56]. In the long run, mutations are predestined to be selectively eliminated if detrimental, or accumulated and fixed via random drift. These processes have consequences for population evolution ranging from the regeneration of the total genetic pool to extinction.

In this report, the percentage of deleterious lethal phenotypes varied between the samples of specimens analyzed, with higher rates occurring in the CdS (6/10) and VC (8/16) harbour sites. In contrast, non-lethal alterations were largely dominant in FuI (9/11). Difference in mutant frequency correlated only between VC/CdS and FuI populations at a reduced geographic scale (tens of Km). This, together with the elevated migration rate between the CDS and the VC population, the results suggest that local biogeographic constraints do not contribute to isolation by distance in open water habitats. The genetic isolation of the FuI population and the predominance of early non-lethal mutant phenotypes indicated that the genetic variants responsible for the phenotypic variation are maintained due to population isolation. In this situation, the dispersal capabilities of larvae may play an important role. Whereas *C. intestinalis* larvae attach to the substrate shortly after hatching [7], uncovered non-deleterious traits do not influence dramatically the overall fitness of the individual and they behave as nearly neutral mutations.

Although microsatellite loci were generally in accordance with HW equilibrium for VC and CdS sites, the FuI population deviated from expectations in the majority of the loci when significance values were combined across them. Here, we attribute this deviation to a non-random mating among individuals followed by inbreeding and natural selection rather than the result of an excess of heterozygotes. The predominance of non-lethal phenotypes in the FuI population supports this view. Non-random mating and inbreeding, when outcrossing rates are low to absent, are able to increase the frequency of homozygous genotypes and therefore eliminate large part of deleterious mutations that are not completely dominant. Individuals carrying highly heritable deleterious allelic variants are hence quickly eliminated by natural selection, which is further supported by environmental quality and features. On the other hand, CdS and VC populations occupy open, circulating coastal waters and they display high levels of gene flow and predominant rates of lethal alterations (Table 4). We assume that outcrossing levels, due to the observed immigration of genotypes between the two sites and the nearby populations, appear to be far higher. Strong outcrossing rates reduce the drastic selective effect of environmental stressors in less dynamic habitats by increasing the within-individual heterozygosity levels. The resulting population genetic architecture is characterised by genetic homogenization in small geographical scale, higher individual fitness, increased heterozygous mutation load in the population and local gene flow. These settings prevented from microsatellite-detectable genetic separation among sub-groups of individuals independently of adaptation attempts to local eco-physiological constraints [57]. FuI individuals inhabit the inner side of a closed lagoon, a polluted ecosystem that communicates with the open sea through three narrow channels. The FuI population appears more exposed to environmental selection and is probably utilizing the genetic variation found here to minimize the effects of negative selection. This population occupies specific microhabitats and, as in VC and CdS, it oscillates between periodical extinction events from the upper layers and subsequent recolonization by deeper refugees [25]. These environmental constraints coupled with non-random mating, or eventually selfing, offer the possibility of reproductive assurance of selected mating combinations and an increased probability that locally adapted genotypes are able to persist [2].

It is known that isolated populations are susceptible to genetic changes at higher rates. Moreover, genetic alterations promoted by pollutants have been inferred by means of molecular markers [58]. Yet, the distinction between a) population genetic structuring according to biogeographical expectations and b) genetic variation accumulated by the influence of ecotoxicological stressors has been investigated for only few species [56].

Current evidence suggests that mutations in key developmental genes that control morphogenesis can cause morphological discontinuities with variable levels of fitness, and that the selective advantage of such variation is primarily filtered by the ecological context. In *Ciona* populations, high levels of genetic diversity may derive from several factors, including the mutation-selection balance between the reappearing phenotypes and their elimination by natural selection, and the population genetic structure driven by reproductive strategies, geographical connectivity and effective population size. In this report, we observe a high load of recessive alleles that affect distinct cell types, tissues and organs. These phenotypes are probably ephemeral entities with low fitness that contribute to local adaptation and do not represent potentially independent evolutionary lineages but are rapidly eliminated. This hypothesis is supported by the reduced overall morphological complexity of the species in an ecological scenario characterized by low intraspecific competition and high physical stress. Alternatively, these loci could represent young, deleterious and spatially restricted alleles with evolutionary potential.

Tunicates exhibit lineage-independent changes in developmental processes among closely related taxa, as shown by the diversity of sensory organs, tail, adhesive organs and epidermal ampullae at the larval stage [59]–[62]. We identified a suite of recessive phenotypes of *C. intestinalis* characterized by morphological alterations that are strikingly reminiscent of the larval anatomy in species belonging to different ascidian families. In tadpole larvae, the putative homologue of the vertebrate fore- and midbrain is the sensory vesicle, a structure that usually contains two sensory organs, the ocellus and the otolith, which are sensitive to light and gravity, respectively [43]. It is thought that important structural changes in sensory organs of ascidians could occur rapidly. Indeed, these organs differ in terms of presence, position, size and structure across the ascidian phylogeny [59]–[61]. In *C. intestinalis* larvae, the frequency of mutations displaying loss, size alteration or supranumerary sensory organs provides a number of phylo-mimicking phenotypes that may be used to uncover the changes in regulatory gene networks that underlie the morphological diversity. For instance, the absence of an ocellus in *omero* and *tasso* phenotypes is typical of many Molgulid species. Similarly, lack of an otolith in the larvae of Clavelinid taxa (*Pycnoclavella* spp.) is evocative of the abnormal sensory organs of *pascoli* and *omero* (Figure 1) [60], [61].

The fact that defects of sensory organ (and tail) differentiation are non-lethal is not surprising if one considers the independent evolutionary loss of these organs in different ascidian taxa. We postulate that such changes in sensory input are related to colonization of altered habitats. It is notable that abnormal phenotypes of the two brain sensory structures occur without correlated changes in other developmental programs and, as in the cases reported in this study, may be considered to be adaptations in a highly polymorphic species [59]–[62].

Spatial structuring of interconnected populations and the distribution of mutant classes follow biogeographical expectations. Whether the impact of pollutant stressors is discernable in *C. intestinalis* at the population level, or it is obscured by random genetic drift, immigration and negative selection, still remains to be clarified. Indeed, we do not exclude the possibility that genetic patterning as well as frequency distribution of deleterious alleles may be selectively promoted by environmental stressors in dynamic habitats of extreme anthropogenic impact such as those analyzed in this study. The combination of marker-assisted analysis of population structure and screening of phenotypic classes is central not only in shaping models in developmental biology but also in evolutionary genetics of populations. The comparison between frequencies of natural variation and population genetic patterning represents a unique opportunity to quantify the importance of biogeographic and anthropogenic factors in affecting mutation–selection dynamics.

### Conclusions

Although the evolutionary population biology of *C. intestinalis* is still scarce, short generation time, strong genetics and genomics, mode of reproduction, closely related sub-populations with high rates of genetic variance, and ways of retaining frozen stocks of genetic lines make this species a model with a future in evolution studies in the wild.

**Figure 6:**
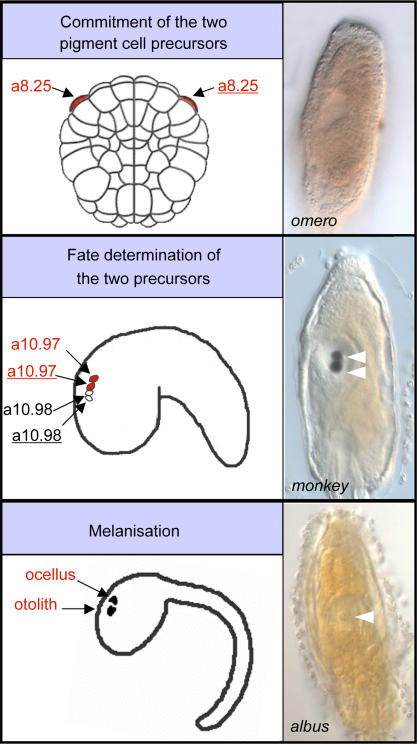
A schematic representation of sensory organ formation in C. intestinalis. (A) Otolith differentiation in *omero* is affected by an early specification problem that is likely to occur during gastrulation. (B) A supranumerary pigment cell in the *monkey* otolith points to a defect in fate determination at the tailbud stage. (C) Pigment formation in the sensory organs implicates a late differentiation step that becomes obvious at the late-tailbud stage, and is abolished in *albus*.

The high mutation frequency revealed in this study indicates that direct functional studies may be carried out in *Ciona* once the gene responsible for the phenotype is identified. The occurrence of abnormalities in early determination, cell fate and differentiation of sensory organs in *C. intestinalis* populations may disclose hidden aspects of the genetic hardwiring (Figure 6), and suggest comparative views on the molecular networks that control differentiation and growth in chordates.

## Materials and Methods

### Sampling and Screening

Specimens of *C. intestinalis* sp. A were sampled from three sites along the southern Thyrrenian coasts of Italy (Mediterranean Sea), two located in the harbours of Villaggio Coppola (VC) and Castellamare di Stabia (CdS), and one in a brackish water lagoon, the Lake Fusaro (FuI). Wild-caught animals were maintained in 35 lt tanks with flow-through seawater and fed as previously described [63]. After two-three days, ripe ascidians were placed under constant illumination for three days in order to trigger gamete maturation. Then, 5–10 specimens were placed in 0.5 l dark chambers containing 0.22 µm filtered seawater (FSW) for 30 minutes as a means of inducing spawning of both homotypic gametes. Released eggs were numbered, maintained at 18°C in FSW (max. 200–300 eggs *per* 15 cm Petri dish) and monitored for cell division in order to measure self-fertilization rates. Only batches containing at least 30 developing embryos were kept for further observations. Embryos were separated from unfertilized eggs and grown at 18°C in FSW. At the larval stage (18 hours post fertilization), inbred offspring were visually scored for defective phenotypes by stereomicroscopy. Behavioral and neuro-muscular abnormalities were inspected by analysis of larval tail movements. Candidate heterozygote carriers of mutant alleles affecting 20–30% of the embryos were left to rest for two weeks before repeating the self-fertilization test. Unfit carriers (siphon closure, body shrinking) were anesthetized in order to collect sperm from the *vas deferens* with a Pasteur pipette. In this and in the case of confirmed carriers, sperm were outcrossed with wild-type eggs (of the same genetic background) for inheritance tests and/or cryopreserved. When individuals carried a limited amount of sperm, male gametes were used solely for outcrossing. Resulting F_1_ offspring were genotyped at the larval stage for mutant allele transmission by self-fertilization tests. Limited storage space and information from re-selfing limited the number of outcross populations for transmission analysis to 11 lines, while complementation tests of similar mutation phenotypes were not performed at this juncture.

### Microsatellite Screening & Population Genetic Analysis

Total genomic DNA was extracted as previously described [18] from 60 randomly sampled *Ciona intestinalis* A individuals (20 from each locality *per* population). Specimens were multilocus genotyped with 4 previously reported [21] and 8 newly selected microsatellite markers [64]. Allele detection was conducted using an automated capillary CEQ 2000XL DNA Analysis system (Beckman Coulter; http://www.beckmancoulter.com) and electropherograms were analyzed with the Beckman CEQ 2000 v3.0 software. Quality of the allelic matrices and post-genotypic errors by population and locus were assessed in MICRO-CHECKER v2.2.1 [65]. Allelic richness *per* locus *per* population, partition of the genetic diversity and variation, gene-flow, F-statistics, number of migrants, analysis of molecular variance and factorial correspondence analysis of the microsatellite data, were explored as implemented in GENALEX v6 [66]. Finally, deviation from Hardy-Weinberg equilibrium was estimated in GENEPOP v3.4 [67], from the 12 microsatellite allelic frequency data, among VC, CdS and FuI populations.

### In situ hybridization

Riboprobe preparation: cDNA clones were identified via bioinformatic screening of a *C. intestinalis* cDNA library (http://ghost.zool.kyoto-u.ac.jp/indexr1.html). Clones of interest were isolated and sequenced using a 3730 DNA Analyzer apparatus (Applied Biosystem; http://www.appliedbiosystems.com). Sequence validation was performed by alignment with the *C. intestinalis* genome (http://genome.jgi-psf.org/ciona4/ciona4.home.html). RNA probes were synthesized using the DIG RNA labeling kit (Roche; https://www.roche-applied-science.com). Whole mount *in situ* hybridization was performed as previously reported [68]. The corresponding cDNA clones used for riboprobe synthesis were *Ci-opsin1* (ID: R1CiGC28m24), *Ci-bmp5*/7 (ID: R1CiGC04j07), *Ci-MITF* (ID: R1CiGC28k08) and *Ci-tyrosinase* (ID: R1CiGC33c19). Images of embryo were recorded using an AXIO Imager.M1 microscope connected to an AxioCam HRc camera (ZEISS; http://www.zeiss.com/) and elaborated with Adobe Photoshop v7.0 or vCS.

### Swimming assays

To study in detail the defect that caused *curly* mutants to continue circling for 24 hours, electrophysiological recordings were made from tail muscles as previously described [69]. Glass micropipettes were drawn from borosilicate glass of 1.5 o.d. on a microelectrode puller (Model P87, Sutter Instrument Company; http://www.sutter.com). The electrodes were mounted on a micromanipulator and their tips broken under microscopic control, so that the internal diameter was about four-fifths the diameter of the larval tail. Using coarse manipulation of the microscope stage and micromanipulation of the electrode, the larval tail was placed in close contact with the tip of the electrode. Then, negative suction was applied and the larval tail was drawn into the pipette to about two thirds of its length. Muscle action potentials were then recorded differentially between the inside of the pipette and the seawater of the bath, and amplified (WPI model DAM 80 World Precision Instruments Ltd; http://www.wpiinc.com) 10000 times with reference to a silver chloride pellet placed in the bath. Signals were AC-coupled, passed between 0.1 Hz and 10000 Hz and digitized and stored, using a Digidata 1200 data acquisition system. Analysis was carried out with Clampfit v9.0 software (Molecular Devices; http://www.moleculardevices.com). A custom-built shutter was controlled by 5 V pulses delivered from the Digidata board, allowing a step-down in the light intensity to be applied for 3 s.

All experiments were carried out at 20°C and preparations were continuously superfused with FSW (8 ml/min^−1^). Larval activity was recorded in a series of 1 min sweeps, one every 5·min, under constant light conditions with a 3 s light-off stimulus to test the shadow response. Plots of instantaneous frequencies of potentials *vs* time and mean frequency of potentials were obtained from raw traces. The duration of each interval of larval swimming activity, also termed ‘burst’, and the quantity of activity for each sweep (sum of all burst durations), both with light-off stimulation and in constant light, was obtained. Recordings were made from newly hatched larvae and from larvae up to 6 hr post-hatching. The activity of each larva was recorded for a maximum period of 3 h.

### Drugs, compounds and solutions

Freshly 0.22 µm filtered sea-water was continuously perfused over the preparation at a rate of 8 mL/min. CNQX was purchased from Tocris (http://www.tocris.com) and added prediluted from a concentrated stock solution and to the superfusate at the final concentrations indicated.
